# Left ventricular endocardial ecchinococcosis associated with multiple intracranial hydatid cysts

**DOI:** 10.1186/1749-8090-8-104

**Published:** 2013-04-20

**Authors:** Ahmad K Darwazah, Mahmoud Zaghari, Mohammed Eida, Mohammed Batrawy

**Affiliations:** 1 Department of Cardiac Surgery, Ramallah Hospital, Ramallah, Israel; 2 Department of Cardiology, Ramallah Hospital, Ramallah, Israel; 3 Department of Cardiac Surgery, Ramallah and Makassed Hospital Mount of Olives, Jerusalem 91194, Israel

**Keywords:** Hydatid disease, Left ventricular ecchinococcosis, Intracranial hydatid cysts, Albendazole, Surgical excision, Endocardial hydatid cyst

## Abstract

Cardiac ecchinococcosis is a rare disease. Its incidence varies from 0.02-2%. Commonly seen in the left ventricle arising from the myocardium in the subepicardial region.

We report a 15-year-old boy presented with a rare combination of a left ventricular subendocardial hydatid cyst associated with multiple cysts in the left cerebral hemisphere and right posterior occipital lobe. The patient underwent successful surgical excision of the left ventricular hydatid cyst using cardiopulmonary bypass.

## Background

Cardiac ecchinococcosis is a rare disease. The heart is commonly involved as a part of systemic infection. Rarely, it can occur as an isolated lesion [1]. The left ventricle is the commonest area affected followed by right ventricle, interventricular septum, left atrium, right atrium and interatrial septum [1].

The combination between cardiac and cerebral hydatid disease has been reported previously [2-6]. In a few cases [3,4], evidence of embolization to the brain from cardiac lesions was documented. While, in others no evidence was found [2,5].

We present a case of multiple cerebral hydatid cysts associated with intracavitary left ventricular hydatid cyst. Management as well as the difficulties encountered during surgery are discussed.

## Case presentation

A 15-year-old caucasian male patient was transferred urgently for surgical management of a left ventricular cardiac mass.

The condition started a year before, when the patient had a seizure which was diagnosed as epilepsy. The patient received Epanutin and remained well and active without any neurological symptoms.

**Figure 1:**
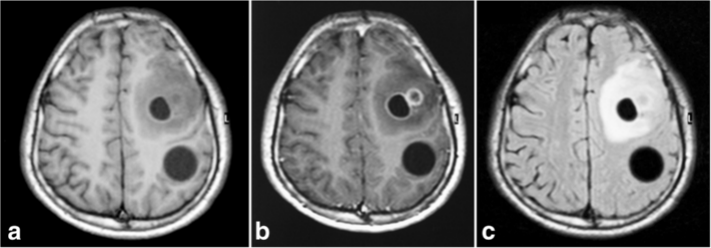
**Brain MRI.** Pre (**a**), Post contrast (**b**), T1 Weighted images and FLAIR (**c**) sequences showing multiple intraparenchymal brain cysts associated with perilesional edema.

Two weeks before admission, he had repeated attacks of eight consecutive seizures. An urgent brain MRI showed multiple, well defined, capsulated cystic lesions in the left cerebral hemisphere and right posterior occipital lobe (Figure 1). On searching for other associated lesions by chest X-ray and abdominal CT, solitary cystic lesions were found in the spleen and right kidney.

The patient was diagnosed with hydatid disease on the basis of being endemic in the area. He was given Albendazole 800mgm and Epanutin 300mgm per day and was prepared for brain surgery.

During preparation, abnormal electrocardiographic changes were detected in the form of inverted T wave in L1, aVL, V4-V6. Surgery was postponed for further cardiac evaluation.

Transthoracic echocardiography showed a 4.5 × 4 cm left ventricular mass. There was mild regurgitation of the mitral valve with no evidence of chamber enlargement or obstruction. Left ventricular function was well preserved.

On admission, the patient was active and cooperative. Clinical examination was unremarkable. Laboratory investigations were normal. Serology for ecchinococcus antibodies was negative.

**Figure 2:**
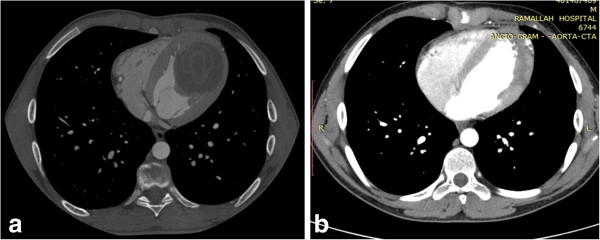
(a) Preoperative cardiac CT showing a left ventricular cystic mass with snake or serpent sign; (b) Postoperative CT showing no recurrence of the cyst during follow up.

Confirmation of a subendocardial left ventricular mass was made by cardiac CT (Figure 2a).

**Figure 3:**
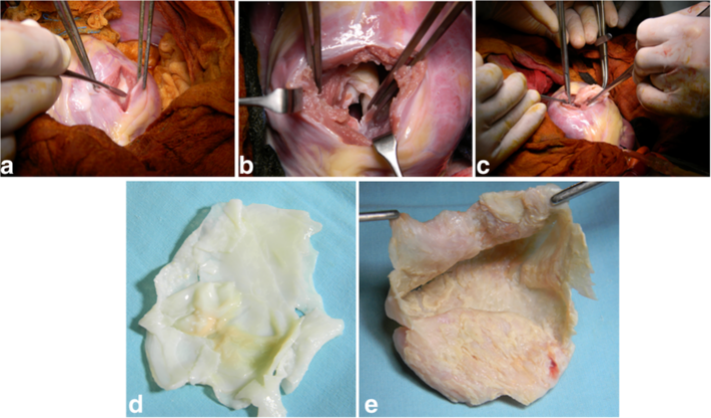
**(a, b, c) Left ventriculotomy incision showing excision of a whitish cyst.** It is formed of a germinal membrane (**d**) and a thickened fibrous capsule (**e**).

**Figure 4:**
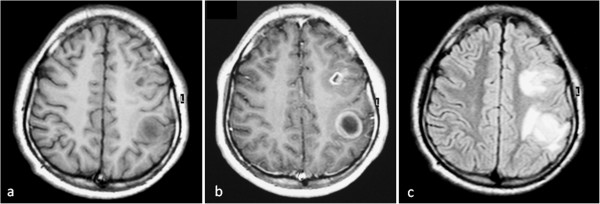
**Brain MRI showing reduction of the size of the cysts and perilesional edema after receiving Albendazole.** Pre (**a**), Post contrast (**b**), T1WI and FLAIR (**c**) showing reduction of the size of the lesions and perilesional oedema after 3 months of treatment. The size of the largest lesion is 2 cm in diameter.

Surgery was performed through median sternotomy. Standard CPB with systemic hypothermia (32-34°C) and antegrade cardioplegia were used. The surgical field was covered with swabs soaked with concentrated saline. The cyst was exposed through left ventriculotomy which appeared whitish and adherent to the under surface of the myocardium (Figure 3a,b). Aspiration revealed a yellowish, thickened material which was difficult to evacuate. The mass was opened and all content was evacuated with complete removal of germinal membrane (Figure 3c,d,e). The cavity was cleaned with concentrated saline. The cyst was adherent to the lateral, posterior, apical and septal area. It was sharply dissected and removed. During dissection, the lower part of interventricular septum was injured and subsequently repaired by using PTFE patch. The whole cyst was excised except for a small portion covering the posteromedial papillary muscle. Left ventriculotomy was repaired by interrupted Teflon pledgets sutures. Weaning from bypass was easy without any support. Postoperative TEE showed no evidence of mitral regurgitation or shunting across the interventricular septum. Histopathology of the excised cyst showed an inner germinal layer surrounded by fibrous capsule with occasional scolices. Follow up by cardiac CT showed no evidence of recurrence (Figure 2b). Perilesional oedema and the size of cerebral cysts were reduced as shown by MRI (Figure 4).

### Discussion

The left ventricle is the most frequent site involved in cardiac ecchinococcosis with an incidence of 55-60% [7]. The larvae of ecchinococcus granulosus reaches the heart via coronary circulation or pulmonary veins [8]. These larvae grow slowly forming a mature cyst. These cysts are usually located in the subepicardial region. Rarely, they appear in the subendocardial area with intracavitary expansion [8].

Clinical presentation is variable depending upon location, size and the involvement of surrounding structures. Due to slow growth of these cysts, patients are often asymptomatic and present in adulthood.

Cysts located in the subepicardial region may compress coronary arteries causing chest pain [9]. They may rupture into pericardial cavity causing pericarditis, tamponade, anaphylaxis and death [7,9]. Those arising from the subendocardial area may rupture causing severe anaphylactic reaction, death and peripheral embolism [7]. Intracavitary growth may interfere with valve function causing stenosis or incompetence [9]. Involvement of the interventricular septum may interfere with the conduction system leading to rhythm disturbances [9].

Similar to cardiac ecchinococcosis, involvement of the brain is rare with an incidence of 1-2% [2]. It is commonly seen in children, presenting with symptoms of increased intracranial pressure associated with various neurological manifestations [6].

In the majority of cases, the brain is involved directly by larvae forming a primary cyst which is usually solitary and located in the supratentorial region in the parietal lobe [2]. Less commonly, the brain is affected by multiple cysts which develop either from spontaneous, traumatic or surgical rupture of a primary solitary cyst or from rupture of an extracranial hydatid cyst which embolises to the brain [10].

Our young patient presented with neurological manifestation in the form of seizures which completely masked the cardiac symptoms despite the fact that the left ventricular cyst was of considerable size. The suspicion of having a cardiac lesion was obtained from electrocardiographic changes which were non-specific.

The presence of multiple brain hydatid cysts in our case most probably denotes that they are secondary in nature. Our patient had no history of trauma or brain surgery. In addition, there was no history of anaphylaxis thus the possibility of being primary is excluded specially in the presence of hydatid disease elsewhere including the heart, kidney and spleen.

The association between cerebral and cardiac hydatid disease has been reported previously [2-6]. Under rare circumstances, multiple cerebral hydatid cysts may develop secondary to embolisation as a sequelae to rupture of an intracardiac hydatid disease [3,4]. In other cases [2,5], the association was found without evidence of rupture of an intracardiac lesion.

In our case, there was no evidence found during surgery denoting the presence of rupture of cardiac cyst.

Management of cardiac hydatid cyts includes both medical treatment and surgical intervention.

Medical treatment in the form of antiparasitic agent (Albendazole) is used as a sole treatment in small calcific cysts especially in asymptomatic elderly patients without hemodynamic effects [11]. These agents are also used both pre and postoperatively to reduce parasitic growth to avoid dissimination during surgical excision and to prevent recurrence.

Medical treatment was an important issue in our patient to control brain cysts. We believe that brain surgery was not applicable in the present circumstances due to several factors including the associated cardiac lesion, multiplicity of cerebral cysts and the presence of active inflammation. The effectiveness of medical treatment was well demonstrated during follow up. The perilesional edema as well as the size of cerebral cysts were reduced. The patient remained asymptomatic with no evidence of new neurological manifestations.

The primary treatment of left ventricular hydatid cyst is surgical excision [7]. Cysts located in the anterior wall and apical region in the subepicardial area usually grow outwards towards pericardial cavity. These cysts are approached directly on beating heart. The cysts are removed leaving the outer fibrotic layer, which can be either left open or obliterated [7]. Cysts arising from anterolateral or apical wall with intracavitary extension as in our present case are approached through left ventriculotomy [7]. The whole cyst with its fibrotic wall should be excised completely with the aid of cardiopulmonary bypass.

Various challenges were encountered during management of our case. There was great concern about the presence of perilesional oedema around cerebral cysts. Every effort was made to avoid brain oedema during cardiopulmonary bypass. The combination of ultrafiltration, diuretics and steroids were used during bypass.

The outer fibrotic layer of the cyst was densely adherent to the inner surface of LV cavity at the region of the septum. Dissection along the septal region resulted in perforation of part of interventricular septum. Excision of the fibrotic membrane was not totally performed to avoid compromising the mitral valve.

## Conclusions

Excision of left ventricular intracavitary hydatid cyst using CPB in the presence of multiple active brain hydatid cysts is safe. Great precautions must be taken during dissection of the cyst to avoid injuring neighbouring structures.

## Consent

Written informed consent was obtained from the patient for publication of this case report and any accompanying images. A copy of the written consent is available for review by the Editor-in-Chief of this journal.

## Competing interests

The authors declare that they have no competing interests.

## Authors’ contributions

All authors were involved in the management of the patient. AKD carried out the operation and writing the manuscript. MZ and ME assisted during surgery and follow up of the patient. MB investigated the patient. All authors read and approved the final manuscript.
